# Toxicity of stainless and mild steel particles generated from gas–metal arc welding in primary human small airway epithelial cells

**DOI:** 10.1038/s41598-021-01177-7

**Published:** 2021-11-08

**Authors:** Andrea Cediel-Ulloa, Christina Isaxon, Axel Eriksson, Daniel Primetzhofer, Mauricio A. Sortica, Lars Haag, Remco Derr, Giel Hendriks, Jakob Löndahl, Anders Gudmundsson, Karin Broberg, Anda R. Gliga

**Affiliations:** 1 Institute of Environmental Medicine, Karolinska Institutet, Box 210, 171 77 Stockholm, Sweden; 2 Department of Organismal Biology, Uppsala University, Uppsala, Sweden; 3 Ergonomics and Aerosol Technology, Lund University, Lund, Sweden; 4 NanoLund, Lund University, Lund, Sweden; 5 Department of Physics and Astronomy, Applied Nuclear Physics, Uppsala University, Uppsala, Sweden; 6 The Tandem Laboratory, Uppsala University, Uppsala, Sweden; 7 Department of Laboratory Medicine, Karolinska Institutet, Stockholm, Sweden; 8 Toxys, Leiden, The Netherlands

**Keywords:** Cancer, Health occupations

## Abstract

Welding fumes induce lung toxicity and are carcinogenic to humans but the molecular mechanisms have yet to be clarified. The aim of this study was to evaluate the toxicity of stainless and mild steel particles generated via gas–metal arc welding using primary human small airway epithelial cells (hSAEC) and ToxTracker reporter murine stem cells, which track activation of six cancer-related pathways. Metal content (Fe, Mn, Ni, Cr) of the particles was relatively homogenous across particle size. The particles were not cytotoxic in reporter stem cells but stainless steel particles activated the Nrf2-dependent oxidative stress pathway. In hSAEC, both particle types induced time- and dose-dependent cytotoxicity, and stainless steel particles also increased generation of reactive oxygen species. The cellular metal content was higher for hSAEC compared to the reporter stem cells exposed to the same nominal dose. This was, in part, related to differences in particle agglomeration/sedimentation in the different cell media. Overall, our study showed differences in cytotoxicity and activation of cancer-related pathways between stainless and mild steel welding particles. Moreover, our data emphasizes the need for careful assessment of the cellular dose when comparing studies using different in vitro models.

## Introduction

Welding fumes are a mixture of micro- and nanoparticles rich in metals such as Fe, Mn, Ni, Cr and Al. These particles are generated when metals heated above their melting point, vaporize and condense. Exposure to welding fumes has been classified as carcinogenic to humans (class 1, IARC) based on a series of epidemiological studies that reported an increased risk of lung cancer among exposed workers^1^. These studies indicated exposure–response associations increasing with length of exposure as well as total cumulative exposure to welding fumes^2–5^. Particles derived from stainless steel welding contain Ni and Cr, and have been reported to be more reactive and toxic in vitro and in vivo than particles derived from mild steel welding, which contain mostly Fe and Mn^6–8^. However, based on epidemiological data, all exposures to welding fumes increases risk of lung cancer regardless of the type of steel welded (mild or stainless), welding method (arc or gas welding) or co-exposures (asbestos, smoking)^1, 9^. Mild steel welding accounts for up to 90% of all welding and the process using gas–metal arc welding (GMAW) is predominant both in Sweden and Europe (> 50% of total welding)^10^.

The mechanisms behind toxicity and carcinogenicity of welding particles are yet unclear. In vivo studies indicate that exposure to stainless steel welding induced chronic lung inflammation while it was not a potent initiator of lung carcinogenesis in A/J mice, a strain susceptible to tumour formation^11, 12^. However, stainless-steel welding fumes can act as a tumour promoter in two-stage promotion initiation models of lung tumorigenesis (3-methylcholanthrene used as a chemical initiator) in A/J mice^13^. Using a similar experimental model, exposure to mild-steel welding particles also promoted lung tumorigenesis, in the absence of lung inflammation^14^. Other possible mechanisms for tumorigenesis can be via changes in the telomere length. Inhalation of stainless steel welding particles in rats resulted in increased expression of markers of neurodegeneration as well as increase in telomere length in whole brain tissue^15^. Additionally, intratracheal instillation of stainless steel welding particles in rats resulted in an increase in telomere length in peripheral blood mononuclear cells, together with increased markers for oxidative stress^16^. However, human studies reported that mild steel welding is either not associated with changes in telomere length^17^ or associated with shorter telomeres in the peripheral blood^18^.

In vitro studies reveal that stainless steel and mild steel welding particles are cytotoxic, increase the production of reactive oxygen species (ROS) and the release of proinflammatory cytokines (IL-1, IL-6, TNFα), and induce mitochondrial dysfunction as well as lipid peroxidation^7, 8, 19, 20^. These effects appear to be consistently more prominent in cells exposed to stainless steel particles compared with mild steel, a difference attributed to increased content and/or solubility of transition metals e.g. Cr^8, 20^. On the other hand, the effects seen in cells exposed to mild steel seem to be correlated with the size of the particles with an increased effect after exposure to the fine and ultrafine fractions compared to coarse particles^7, 21^.

The aim of this study was to evaluate the acute toxicity of well-characterized mild and stainless steel welding particles smaller than 2.5 µm in primary human bronchial cells from the small airways. The welding particles were generated by gas-metal arc welding with steel electrodes commonly used in occupational settings and were thoroughly characterized from particle generation to cell exposure. In addition, we also used the ToxTracker reporter cells to screen for potentially perturbed pathways related to carcinogenesis.

## Materials and methods

### Particle generation and collection

**Figure 1:**
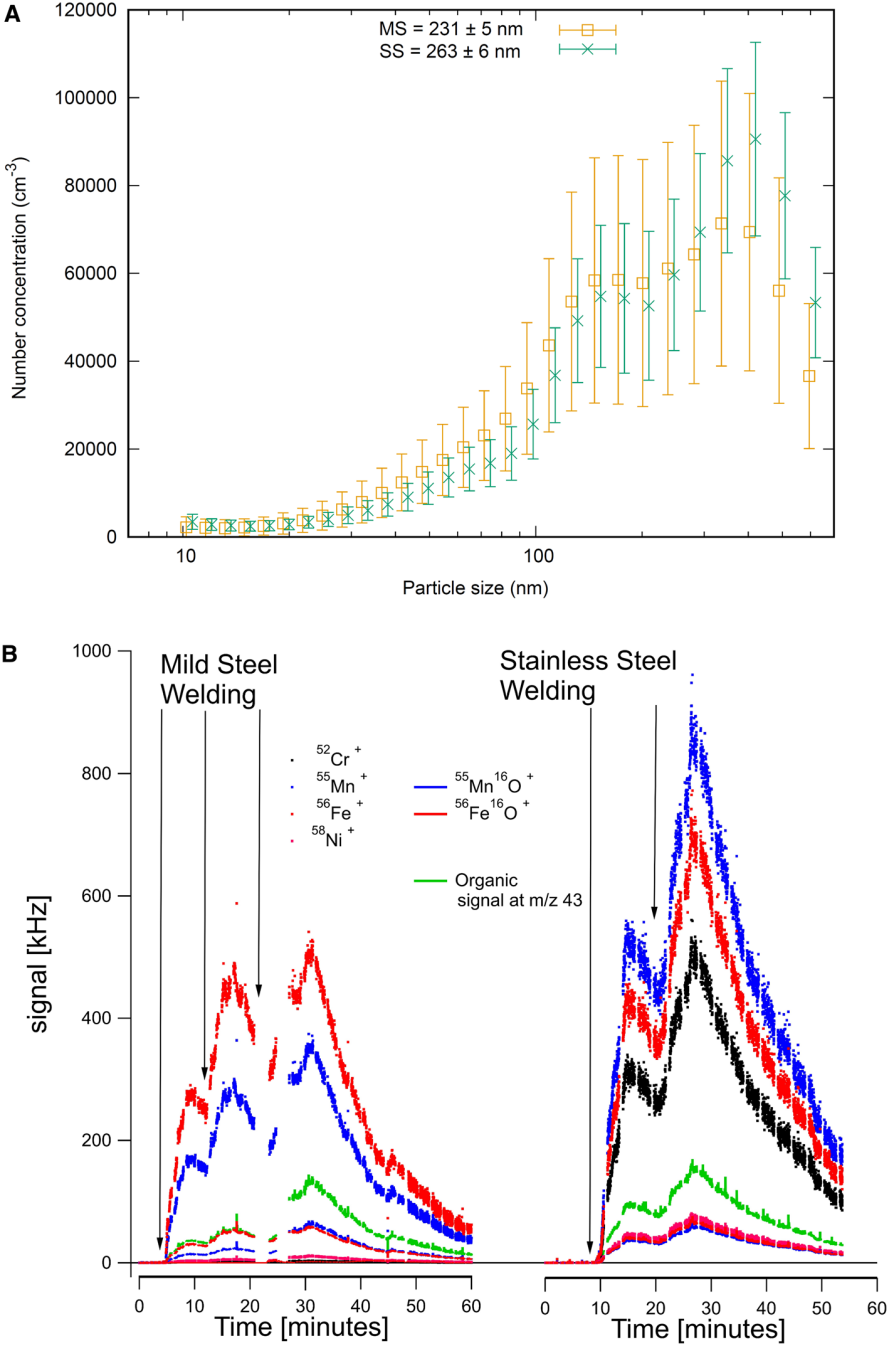
Characterization of welding particles during generation. (**A**) The particle number concentration and mobility size distribution (10–650 nm) were measured using a Scanning Mobility Particle Sizer (SMPS) system. The error bars indicate that welding was not done continuously throughout the sampling period, but in intervals. (**B**) An example of aerosol mass spectrometry spectra. Time resolved aerosol mass spectrometry data showing signal intensity for selected metal and metal oxide ions, and organic signal at m/z 43 (corresponding to 9–11% of total organic signal). Black vertical arrows indicate welding. Figures were created using gnuplot version 5.2 (**A**) and IGOR pro 6.37 (**B**).

Stainless and mild steel particles were generated using gas–metal arc welding (GMAW) in a laboratory setting as previously described^22^. We used two electrodes, Autrod 316LSI (ESAB, Sweden) for stainless steel and Aristorod 12.50 (ESAB, Sweden) for mild steel, selected because they are the most common for the two types of steel in Sweden (personal communication with the European Welding Association and the major electrode retailer). Briefly, the welding was conducted using a Kemppi Kempomig 350 welding system with an electrode feeding rate of 3 cm/s and an 82% Ar 18% CO_2_ shielding gas for mild steel welding and a 98% Ar 2% CO_2_ shielding gas for stainless steel welding. The gas flow rate was 12 L/min. The welding was carried out in pulses of a couple of minutes followed by periods of non-welding (see Fig. 1B for a typical time series). The welding took place in a 1.33 m^3^ volume stainless steel and glass chamber. Using an air amplifier (Coval M10C, Raleigh, USA), the air was transported with an airflow of 500 L/min into a 21.6 m^3^ stainless steel chamber, where the particle collection and aerosol characterization were conducted. Particles < 2.5 µm for the cell studies were collected on PTFE-filters (TE38, 5.0 µm, diameter 150 mm) using a BGI900 high volume sampler (BGI Inc., Waltham, USA) at a flow rate of 900 L/min. Particles were extracted from the filters using pure methanol (MeOH extraction protocol, provided by BGI). After extraction, the particle-MeOH solution was sonicated and pipetted into clean glass tubes and dried using a vacuum centrifuge (SpeedVac HT-4X Evaporator). Particles were also collected for particle induced X-ray emission (PIXE) analysis using a custom built multi stage low pressure impactor (flow rate of 10 L/min and downstream pressure of 0.13 bar), which collects size-segregated samples of particles 0.04–10 µm in 12 stages with aerodynamic cut-off diameters (D50) of: (1) 0.04, (2) 0.09, (3) 0.15, (4) 0.22, (5) 0.36, (6) 0.58, (7) 0.81, (8) 1.07, (9) 1.68, (10) 2.69, (11) 4.46 and (12) 8.55 µm. On each impaction plate the particles were collected on polycarbonate filters covered with a thin layer of grease (Apiezon N grease silicon free).

### Characterization of particles during generation

During aerosol generation, the particle number concentration and mobility size distribution were measured in the range 10–650 nm using a Scanning Mobility Particle Sizer (SMPS) system, consisting of a medium Hauke type differential mobility analyser and a condensational particle counter CPC3010 (TSI, Shoreview, MN, USA) with at an aerosol flow of 1 L/min and a sheath flow of 7 L/min.

Time resolved qualitative composition was measured using a soot particle aerosol mass spectrometer^23, 24^ in “dual vaporizer” configuration. Particles were vaporized with an Nd:YAG laser (λ = 1064 nm) and then ionized with 70 eV electrons followed by time of flight mass spectrometry. The mass spectra were analysed with the software SQUIRREL 1.63I. The large mass defects of the metal and metal oxide ions were exploited to separate their signals by redefining the “stick integration regions” to exclude neighbouring organic ions.

The quantitative metal composition of the welding aerosols was determined by particle induced X-ray emission (PIXE) on the different size fractions. We used a focused 3 MeV proton beam with spatial resolution of 2 × 2 µm (micro-beam), scanning over the sample. The X-rays were detected at 45° with a PGT Si(Li) detector. In addition, samples scraped from the raw electrodes and samples collected on the glass vials were also analysed by PIXE using a 2 MeV broad He beam. For the particles deposited on a glass tube, the bottom of the tube was cut and a piece of the glass containing the particles was measured. A clean piece of the glass was also measured and it contained only traces of K and Ca. The concentrations of elements were obtained by fitting of the experimental spectrum with the software GUPIXWIN^25^. The data for the size fraction 12 (D50: 8.55 µm) was removed from the graphs as the amount of particles in this size range was very low, and there was hence a risk for non-particle interference to take place that would bias the results.

### Preparation of particle dispersions

Welding particles deposited on glass vials were dispersed in endotoxin free water (Sigma, 95289) at a concentration of 1 mg/mL by sonication (Branson 2200) on ice at a frequency of 40 kHz for 25 min (stainless steel) or 60 min (mild steel) to ensure total dispersion (visual inspection). Stock dispersions were freshly prepared before each experiment. For cell exposure experiments, the stock dispersion was diluted in the relevant cell medium.

### Characterization of particles in dispersion

The size distribution of the particles dispersed in water or cell media was obtained by dynamic light scattering (DLS) (Zetasizer Nano-ZS, Malvern Instruments, Worcestershire, UK). 50 µg/mL dispersions were analysed at 25 °C directly after preparation. Data is presented as mean ± SD of dispersions from three different vials of particles for each particle type.

### LAL assay

The endotoxin concentration in the welding particles was determined with the QCL-1000™ endpoint chromogenic Limulus Amebocyte Lysate (LAL) assay (Lonza, 50-647U) performed according to the manufacturer’s protocol. The levels of endotoxin measured in the particle dispersions were below 0.005 EU/mL.

### ToxTracker assay

A panel of six green fluorescent mouse embryonic stem (mES) reporter cell lines developed by Toxys (ToxTracker assay) was used for assessment of the biological damage produced upon exposure to stainless steel and mild steel particles. The ToxTracker assay provides information regarding the activation of genes associated with DNA damage, p53 activation, protein damage and oxidative stress. mES cells were maintained in 60 mm cell culture dishes coated with 0.1% gelatine (Sigma-Aldrich, G1890-100G) using irradiated primary mouse embryonic fibroblasts as feeders, in Knockout DMEM (Gibco, 10829018) supplemented with 10% foetal bovine serum (FBS, QC-ed and certified for ES cells by Toxys), 1X GlutaMAX (Gibco, 35050061), 1 mM sodium pyruvate (Gibco, 11360039), 1X non-essential amino acids (Gibco 11140035), 0.1 mM 2-Mercaptoethanol (Gibco, 31350010), 100 U/mL Penicillin/Streptomycin (PEST, Gibco, 15140122) and leukaemia inhibitory factor (LIF, Toxys custom made). For experiments, mES cells were seeded in 96 well plates (Corning, 3603) coated with 0.1% gelatine at a density of 1.51 * 10^5^ cells/cm^2^ and exposed for 24 h to stainless steel or mild steel particle dilutions at concentrations ranging from 0.5 to 250 µg/mL. After exposure, the cells were washed twice with phosphate buffered saline solution (PBS, Sigma, D8537-500) and harvested. GFP reporter induction was evaluated by flow cytometry with a Guava^®^ easyCyte HT (Merck). Cytotoxicity was evaluated in the same samples by assessing the distribution of the cells in the forward scatter-side scatter plot.

The data is presented as times increase in GFP induction normalized against the negative control (mean ± SD of three independent experiments). An experiment was considered positive if a fold increase of two or more was obtained in the GFP expression.

### Culture of human small airway epithelial cells (hSAEC)

Human small airway epithelial cells (hSAEC, Epithelix, product code: EP61SA) from three donors (2 males, one female, average age 69 years, non-smokers, without any lung pathology) were routinely cultured in 75 cm^2^ cell culture flasks (Life Technologies, 156499) at a density of 9.3 * 10^3^ to 10.6 * 10^3^ cells/cm^2^ in hAEC culture medium. hSAEC were seeded in hAEC culture medium (Epithelix, EP09AM) at a density of 3200 cells/cm^2^ for experiments with 24-h exposure, 2500 cells/cm^2^ for 48 h exposure and 1600 cells/cm^2^ for 72 h exposure. After 24 h, the cells were exposed to stainless steel or mild steel particles and kept in a humidified atmosphere (5% CO_2_ and 37 °C) until the experiments were performed.

### Cell viability

For cell viability assessment, hSAEC were exposed to stainless steel or mild steel particle dispersions at concentrations ranging from 1 to 250 µg/mL and incubated for 24, 48 or 72 h. After exposure, the cell medium in all wells was removed and replaced with a 10% Alamar Blue cell viability reagent solution (ThermoFisher scientific, DAL1025). The cells were subsequently incubated together with this solution for 2 h (5% CO_2_, 37 °C) and afterwards the fluorescence was recorded at 540 nm excitation and 590 nm emission with a plate reader (Tecan infinite F200). A control containing only particles and Alamar Blue reagent (without cells) was included in all plates to evaluate potential particle interference with the assay. Results are expressed as percentage metabolic activity normalized against the corresponding negative control and presented as the mean ± SD of three biological replicates for each donor (n = 9).

### Inductively coupled plasma mass spectrometry (ICP-MS)

The amount of metals released by the particles in the cell medium and the intracellular concentration of metals after exposure to the welding particles were measured by ICP-MS.

#### Sample collection

For evaluation of the amount of metal released by particles, stainless steel and mild steel particle dispersions (50 µg/mL) were prepared in hSAEC or mES cell culture medium. Samples from two time points (immediately after dispersion or after 24 h of incubation at 37 °C) were centrifuged at 40,000 rpm for 30 min (4 °C). After centrifugation, the supernatant fraction was carefully removed and stored together with the pellet remaining fraction at 4 °C for later analysis.

For measuring the intracellular concentration of metals, hSAEC from the three donors were seeded in 6 well plates at a density of 30,000 cells/cm^2^ and wt mES were seeded at a density of 1.1 * 10^5^ cells/cm^2^ in gelatine coated 6 well plates (Life Technologies, 140675). The cells were then exposed 50 µg/mL stainless steel or mild steel particles for 24 h. After exposure the cells were washed thoroughly with PBS, harvested with 200 µL trypsin (Sigma, T4174) at 37 °C for 5 min, collected and resuspended in 200 µL culture media. After harvesting, the cells were counted and kept at 4 °C until processing.

#### Multi-element analysis

The samples were digested in 65% HNO_3_ until visually clear and thereafter diluted to 2% HNO_3_ with deionized water. After digestion, the samples were diluted 1:10 in deionized water and the metals were quantified with iCAP Q (ThermoScientific) in KED mode. The following isotopes were analysed: ^52^Cr, ^53^Cr, ^56^Fe, ^55^Mn, ^60^Ni (the most abundant metals in the particles) and each sample was injected at least 5 times. For the analysis, calibration standards of Cr, Fe, Mn and Ni (0, 1, 5, 10, 50, 100 and 500 ppb, Spectrascan) were prepared in deionized water with 2% HNO_3_. Blank samples containing only cell culture media were analysed together with the particle samples to account for the intrinsic composition of the media. All samples were spiked with 5 ppb In and 5 ppb Rh used as internal standards, the accepted range of internal standard recovery was between 80 and 110%. The limit of detection (LOD) for all metals was below 0.05 ppb.

The percentage of metal released in the media was calculated as the percentage of metal released in supernatant in relation to the total metal content (experimentally determined from the pellet fraction). The metal content of the cells was expressed as pg of metal per cell.

### Cellular localization of welding particles by electron microscopy

Transmission electron microscopy (TEM) and scanning electron microscopy (SEM) were used to evaluate the cellular localization of the welding particles. Cells were seeded in 6 well plates at a density of 30,000 cells/cm^2^ in the case of hSAEC or 1.1 * 10^5^ cells/cm^2^ in the case of wt mES, and exposed to 50 µg/mL of stainless steel or mild steel particles for 24 h. After exposure, the cells were washed twice with PBS, harvested by trypsinization and spun down for 10 min at 1500 rpm. The supernatant was subsequently removed from the pellet and 1 mL of fixative solution (2.5% glutaraldehyde in 0.1 M phosphate buffer pH 7.4) was added. Following fixation, the cells were rinsed in 0.1 M phosphate buffer pH 7.4 prior to post-fixation in 2% osmium tetroxide in 0.1 M phosphate buffer, pH 7.4 at 4 C for 2 h. The cells were then stepwise dehydrated in ethanol followed by acetone and finally embedded in LX-112. Ultrathin sections (60–80 mm) were prepared using an EM UC7 ultramicrotome (Leica) and contrasted with uranyl acetate followed by Reynolds lead citrate. TEM imaging was done in a Hitachi HT7700 transmission electron microscope (Hitachi High-Technologies) operated at 80 kV and digital images were acquired using a 2kx2k Veleta CCD camera (Olympus Soft Imaging Solutions). SEM images were acquired using an Ultra 55 field emission scanning electron microscope (Zeiss) operated at 3 kV and the SE2 detector.

### Generation of reactive oxygen species (ROS)

#### In hSAEC cells

The cell permeant dye 2′,7′-dichlorofluorescin diacetate (DCF-DA, Sigma, D6883) was used for assessment of intracellular ROS production. For experiments, hSAEC were seeded at a density of 3200 cells/cm^2^ in black 96-well plates (Sigma, CLS3603-48EA) and exposed for 24 h to 1, 10, 100 or 200 µg/mL stainless steel or mild steel particle dispersions. After exposure, the cell media containing the particles was removed and replaced with 100 µL of 25 µM DCF-DA. The cells were incubated together with the DCF-DA reagent for 45 min at 37 °C 5% CO_2_. After incubation the DCF-DA was removed and replaced with hSAEC cell culture medium. Fluorescence was measured from the top at 485 nm excitation and 535 nm emission with a plate reader (Tecan F200) every 5 min for 30 min. Tert-Butyl hydroperoxide (TBHP, 200 µM) was used as positive control. The data is presented as times increase in ROS production of the mean slope per minute, normalized against the negative control; mean ± SD of at least two biological replicates for each donor (n = 8).

#### Acellular ROS

To assess the acellular production of ROS, 1500 µL of 50 µM DCF-DA were mixed for 30 min with 6000 µL CH_3_OH and 30 mL NaOH (0.01 M), NaOH deacetylates the DCFDA dye, which is subsequently oxidized into DCF. After mixing, 112.5 mL of 33 mM NaH_2_PO_4_ were added to obtain a 50 µM DCF solution. For experiments 1, 10, 100, 150, 200, 250 or 300 µg/mL stainless steel or mild steel particle dispersions (prepared in 100 µl) were added to a 96 well plate together with 25 µM DCF. Fluorescence was measured from the top at 485 nm excitation and 535 nm emission with a plate reader (Tecan F200) every 5 min for 30 min.

The data is presented as times increase in ROS production of the mean slope per minute, normalized against the negative control (mean ± SD of three independent experiments).

### Statistical analysis

One-way ANOVA followed by Dunnet’s post hoc test (with implicit adjustment for multiple testing) was used for comparisons between treatments and control. For comparisons among treatments un-paired t-test was used. All analyses were performed using GraphPad Prism version 8.3.0 for Windows, GraphPad Software, San Diego, California USA, http://www.graphpad.com. P-values < 0.05 were considered statistically significant.

## Results

### Generation and characterization of welding particles

In this study we generated stainless- and mild-steel welding particles using gas-metal arc welding technique and 2 electrodes (stainless steel, Autrod 316LSI and mild steel, Aristorod 12.50) widely used and relevant for occupational settings in Sweden. The count median diameter of the particle agglomerates during generation was 263 nm for stainless steel and 231 nm for mild steel (Fig. 1A). The mild steel particles consisted mainly of Mn and Fe, while the stainless steel particles were also enriched in Ni and Cr, as indicated by the aerosol mass spectrometry data (qualitative data, Fig. 1B).

**Figure 2:**
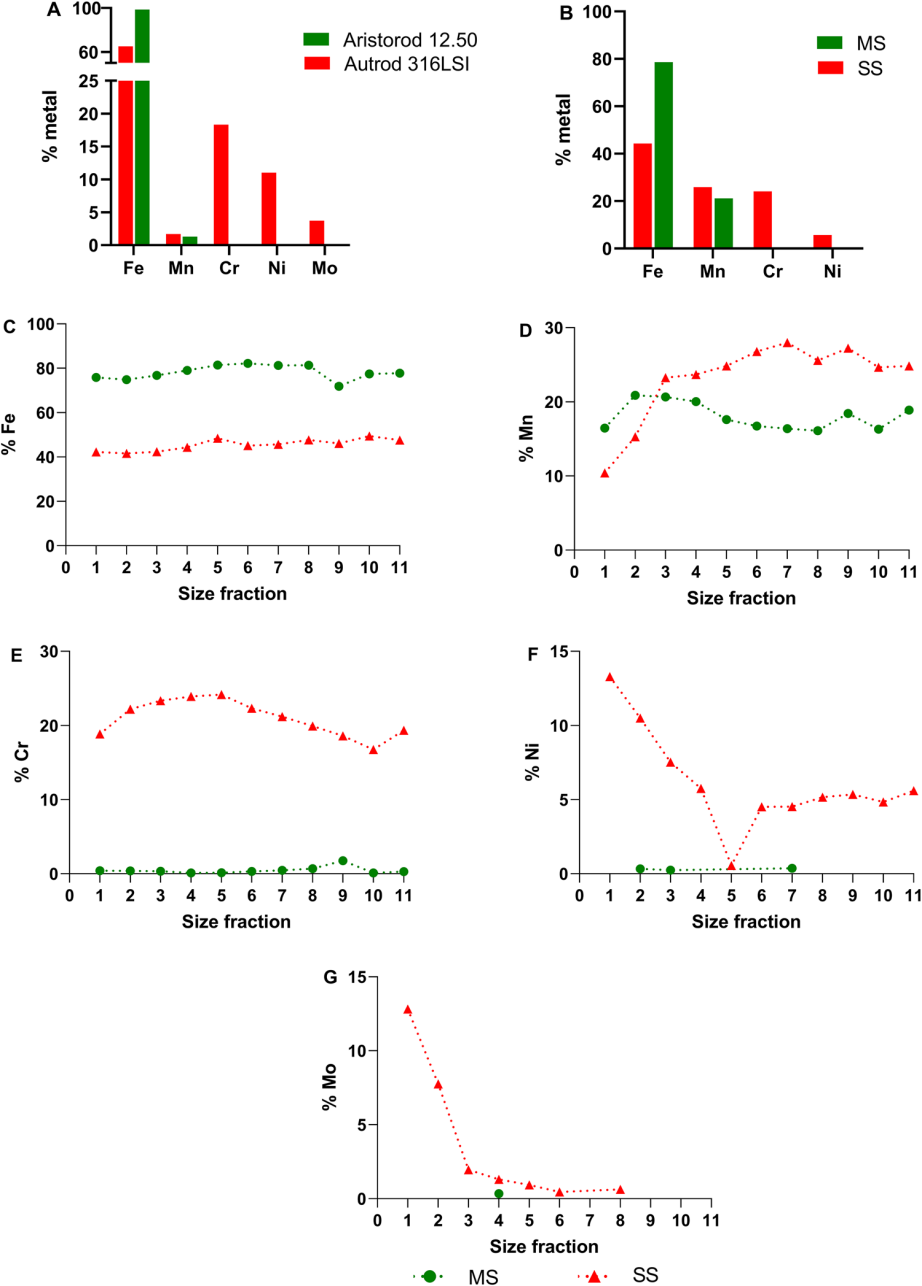
Characterization of welding particles by PIXE. Quantitative metal composition was determined by PIXE on the welding electrodes (**A**), collected particles (**B**) as well as different particle size fractions during the aerosol generation (**C**–**G**). Percentage refers to metal content which was normalized to 100% (*i.e.* it disregards the presence of oxygen). The D50 (µm) for the collection stages are the following: 1—0.04 µm, 2—0.09 µm, 3—0.15 µm, 4—0.22 µm, 5—0.36 µm, 6—0.58 µm, 7—0.81 µm, 8—1.07 µm, 9—1.68 µm, 10—2.69 µm, 11—4.46 µm. Figure created using GraphPad Prism version 8.3.0.

In addition, we performed a quantitative evaluation of the metals by PIXE at different stages: (i) metal electrodes (ii) different size fractions during particles during generation and (iii) collected particles after dispersion in water (Fig. 2 and Supplementary Fig. 1). The results indicate that the mild steel electrode consists predominantly of Fe (98.7%) and small amounts of Mn (1.3%) while the stainless steel electrode contains mostly Fe (65.2%), Cr (18.3%), Ni (11%) and Mo (3.7%), consistent with manufacturer specifications (Fig. 2A). The collected particles contained similar amounts of Mn (25.9% SS and 21.1% MS), while the MS particles were more abundant in Fe (78.6%) compared to the SS particles (44.3%). In addition, and in line with the composition of the electrodes the SS particles also contained Cr (24.1%) and Ni (5.7%) (Fig. 2B). A detailed quantification of the different size fractions indicated that the amount of Fe did not vary within the different fractions for the two particles (Fig. 2C) and were on average 45% for SS and 78% for MS. The Mn content was similar between the size fractions for MS (18% on average) while for SS there was a lower amount of Mn in the smaller size fractions (e.g. 10% in size fraction 1) compared to the larger size fractions (e.g. 28% size fraction 7) (Fig. 2D). SS had a relatively stable Cr content across all size fractions (average 21%) (Fig. 2E), but the Ni content was higher for smaller size fractions (e.g. 13% for size fraction 1 vs. an average of 5% for size fractions 6–11) (Fig. 2F). For SS there was also an enrichment of Mo in the smaller size fractions (Fig. 2G). PIXE data on Cu, Ti and Zn is included in Supplementary Fig. 2. Aerosol mass spectrometry data (not shown) suggests that the metal oxides were also uniformly distributed across all particle sizes, while the organic material present on the airborne particles was shifted towards smaller diameters, consistent with coatings of uniform thickness.

Size of the particle agglomerates in different media (water, mES cell medium and hSAEC cell medium) was evaluated by DLS (Table 1). Directly after dispersion both stainless- and mild-steel particles agglomerated to a higher extent in the serum-free hSAEC medium (mean size: SS 454 nm, MS 346 nm) compared with the serum supplemented mES medium (mean size: SS 242 nm, MS 121 nm). The polydispersity index was higher in mES medium compared with hSAEC medium for both particles which is indicative of a more homogenous dispersion. Surface charge was negative in all environments for both particles.

**Table 1:** Particle characterization by dynamic light scatering (DLS) in water and cell culture media directly after preparation of 50 µg/mL dispersions.

Environment	Stainless steel			Mild steel		
	Z-average (d.nm)	PdI	Z-potential (mV)	Z-average (d.nm)	PdI	Z-potential (mV)
Water	278 ± 75	0.41 ± 0.08	− 20.9 ± 12.4	217 ± 29	0.37 ± 0.05	− 10.7 ± 8.9
mES cell medium	242 ± 33	0.66 ± 0.19	− 10.9 ± 0.5	121 ± 63	0.63 ± 0.24	− 9.7 ± 1.1
hSAEC cell medium	454 ± 209	0.52 ± 0.25	− 11.8 ± 2.1	346 ± 181	0.39 ± 0.14	− 13.3 ± 2.5

Data is presented as mean ± SD.

PdI, polydispersity index; mES, mouse embryonic stem cells; hSAEC, human small air epithelial cells.

**Figure 3:**
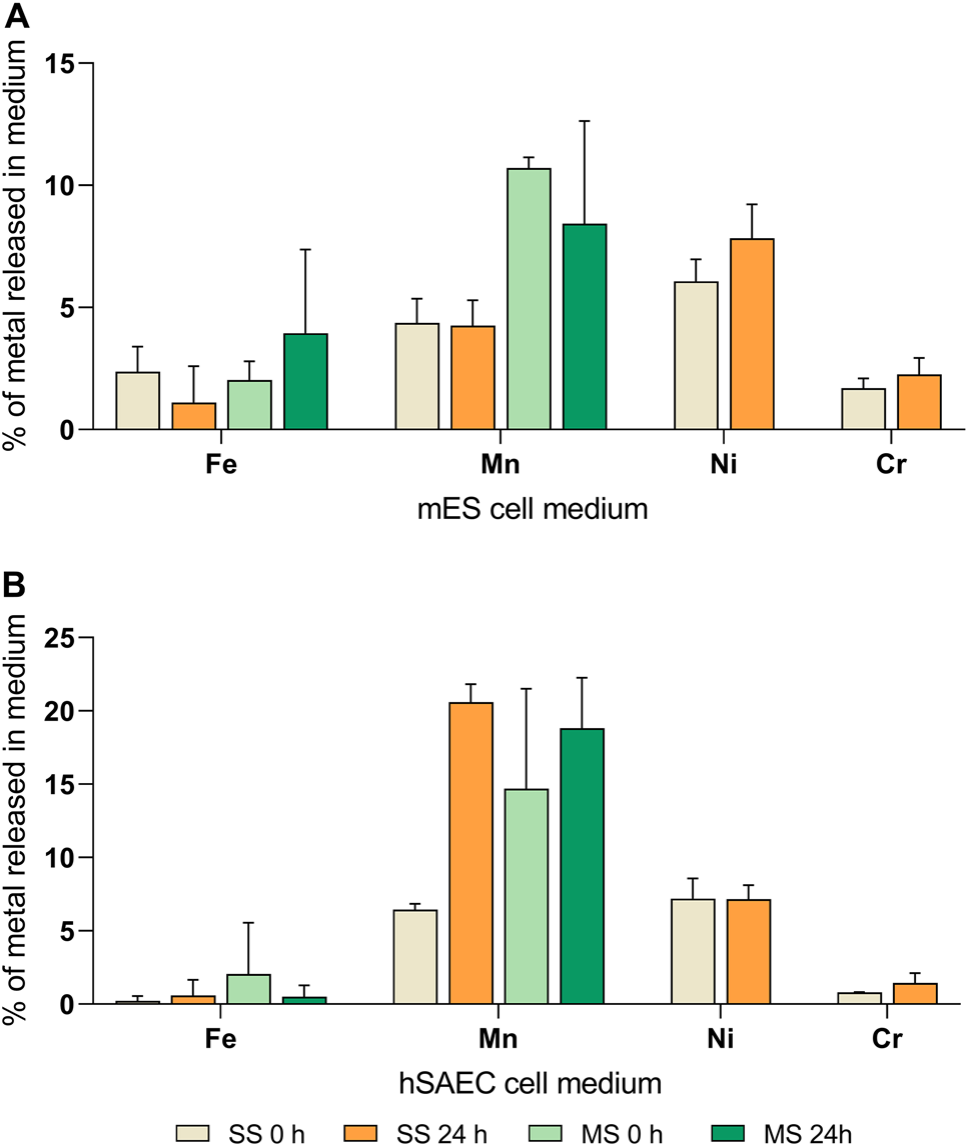
Metal released from welding particles in cell culture media. 50 µg/mL stainless and mild-steel welding particle dispersions were incubated for 24 h with cell medium used for the growth of mES cells (**A**) and hSAEC cells (**B**). The amount of metal (Fe, Mn, Ni, Cr) released in cell medium was analysed by ICP-MS directly after disperson (0 h) and after 24 h. Data was expressed as percentage metal from total metal content in the particles (experimentally determined). Figure created using GraphPad Prism version 8.3.0.

We evaluated the metal fraction released in two types of cell medium (relevant for the cell culture experiments in the present study) right after sonication (0 h) and after 24 h for the most abundant metals i.e. Fe, Mn, Ni and Cr (Fig. 3). In mES medium, Fe was on average under 4% and was similar between the MS and SS particles, while Mn was higher for MS particles (approx. 4% at both timepoints) compared with the SS particles (approx. 11% at 0 h and 8% at 24 h) (Fig. 3A). The Ni released by SS particles was approx. 6% at 0 h and approx. 8% at 24 h while the Cr was approx. 2% at both timepoints in mES (Fig. 3A). In hSAEC medium, the release of Fe was on average under 2% and was similar between the MS and SS particles, while the release of Mn was higher for MS particles directly after dispersion (0 h, approx. 15%) compared with SS particles (approx. 6%) but similar after 24 h for the two particles (21% for SS and 19% for MS) (Fig. 3B). The Ni released by SS particles in hSAEC cell medium was approx. 7% while the Cr released was approx. 1% at both timepoints (Fig. 3B). Interestingly, there was an increase in Mn released in hSAEC medium by the SS particles at 24 h compared with 0 h (Fig. 3B). There were no other time-dependent differences between metal released in the two media for the reported metals (Fig. 3). When comparing the two media for the same particles and timepoints, the metal release was relatively similar for Fe, Ni and Cr, but in general higher in mES for Mn compared with hSAEC (Fig. 3).

### Stainless steel but not mild steel welding particles activate oxidative stress pathways in reporter mouse embryonic stem cells

**Figure 4:**
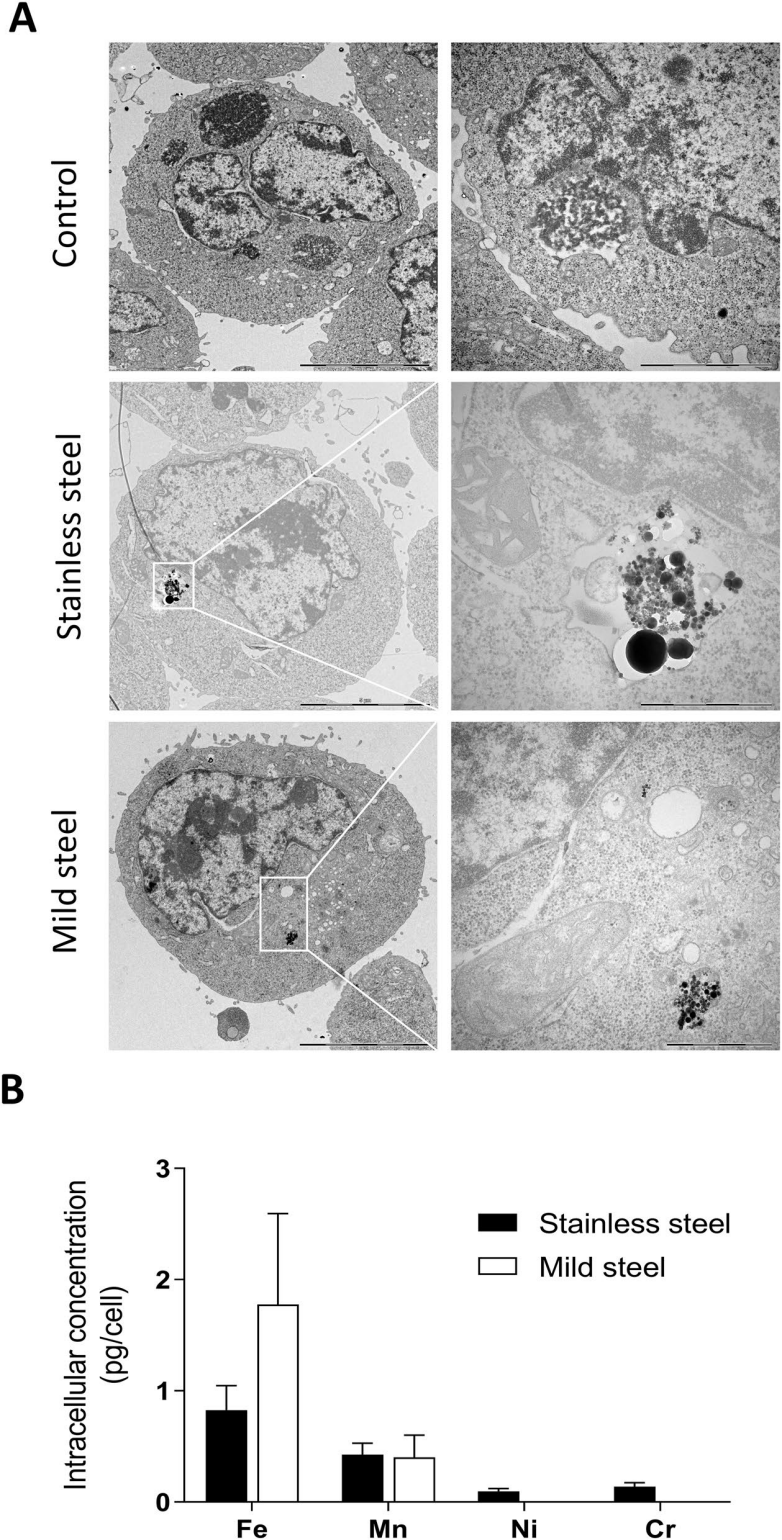
Intracellular localization and metal content of mouse embryonic stem cells exposed to welding particles. The cells were exposed to 50 µg/mL of stainless steel or mild steel particles for 24 h. (**A**) Cellular localization of the particles was confirmed by TEM. (**B**) The intracellular concentrations of Fe, Mn, Ni and Cr were quantified by ICP-MS and the results expressed as pg of metal per cell. Figure 4B was created using GraphPad Prism version 8.3.0.

We used the ToxTracker reporter stem cell assay to gain knowledge on six biological pathways related to carcinogenesis that are activated by exposure to welding particles. First, we confirmed by TEM that the stem cells can internalize welding particles. After cell exposure to 50 µg/mL of SS or MS for 24 h, particles were localized free in the cytoplasm or within membrane-bound structures (endosomes and/or lysosomes) (Fig. 4A). There was no indication of particles present within the nucleus. These observations were complemented with a quantitative analysis of the intracellular metal content (Fe, Mn, Ni and Cr) under the same experimental conditions (Fig. 4B). The results show that cells exposed to MS contained on average approximately double the amount of Fe compared to cells exposed to SS (1.7 pg/cell vs 0.8 pg/cell) and similar amounts of Mn (approx. 0.4 pg/cell). In addition, cells exposed to SS particles also contained Ni and Cr (approx. 0.1 pg/cell). Unexposed cells had levels of Fe and Mn close to the background levels in the cell medium.

**Figure 5:**
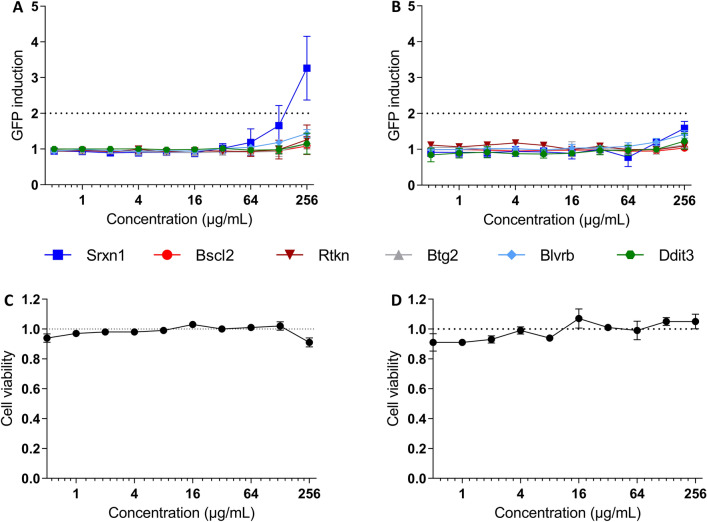
Activation of ToxTracker reporter cells following exposure to welding particles. Six ToxTracker GFP reporter cell lines were used for evaluation of DNA damage (*Bscl2* and *Rtkn*)*,* p53 activation (*Btg2*), induction of oxidative stress (*Srxn1* and *Blvrb*) and protein damage (*Ddit3*). Activation of the reporter cells was assessed by flow cytometry after 24 h of exposure to (**A**) stainless steel or (**B**) mild steel particles (0.5–256 µg/mL). Cell viability was simultaneously measured based on scatter parameters for (**C**, stainless steel; **D**, mild steel). All x-axes are logarithmic (log2). The results are presented as mean ± standard deviation of three independent experiments. Figure created using GraphPad Prism version 8.3.0.

Next, we used the ToxTracker assay composed of six reporter mES cell lines to evaluate reporter activation following exposure to SS and MS welding particles (Fig. 5A,B). After 24 h exposure, SS induced the Srxn1 reporter above the threshold (two-fold change) at the highest concentration tested (256 µg/mL) and there was an activation trend (average above 1.5-fold change) for the second highest concentration (128 µg/mL). This reporter is activated following Nrf2 dependent oxidative stress. There was no effect on the other reporters *i.e.* Bscl2 (DNA replication stress), Rtkn (DNA double strand breaks), Btg2 (p53-mediated stress response), Blvrb (Hmox1 antioxidant responses), DDit3 (unfolded protein response) following SS or MS exposure. Simultaneously to the activation of the reporter cells, the effect of the particles on mES cell viability was evaluated by flow cytometry (Fig. 5C,D); none of the particles negatively affected the cell viability at the concentrations tested.

### Welding particles induce dose- and time-dependent cytotoxicity in primary human epithelial cells from the small airways

**Figure 6:**
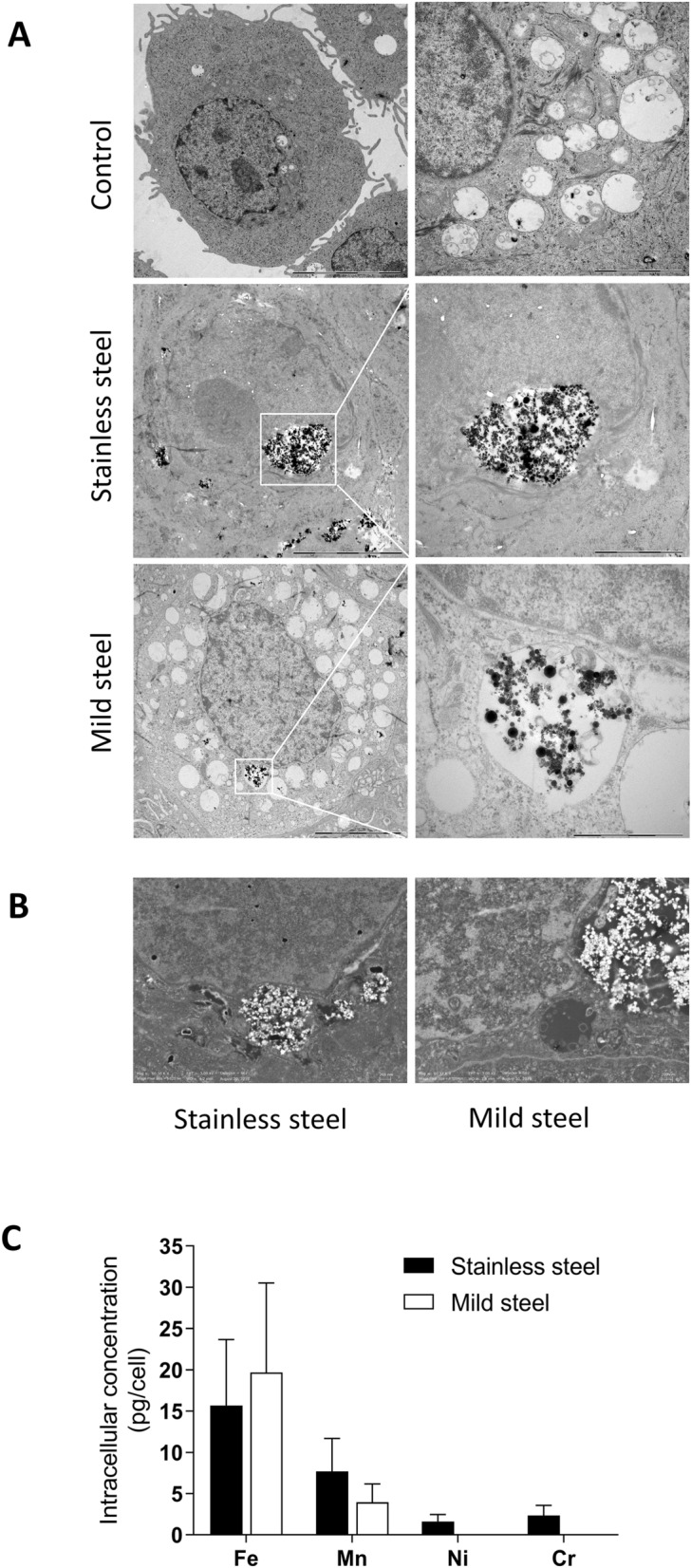
Intracellular localization and metal content of primary human epithelial cells from the small airways exposed to welding particles. The cells were exposed to 50 µg/mL of stainless steel or mild steel particles for 24 h. (**A**) The cellular uptake and intracellular localization of the particles was evaluated by TEM. (**B**) SEM images focused on the intracellular welding particles are included as example. (**C**) The intracellular concentrations of Fe, Mn, Ni and Cr were quantified by ICP-MS and the results expressed as pg of metal per cell. In this figure (**C**) was created using GraphPad Prism version 8.3.0.

Primary human SAEC cells were used as an in vitro model for testing the cytotoxicity of the SS and MS welding particles. Particle uptake and cellular localization by hSAEC was visualized by TEM after exposing the cells for 24 h to 50 µg/mL of SS or MS particles (Fig. 6A). The images confirmed internalization of the particles, which were localized in the cytoplasm, free or within membrane-bound structures. SEM images of the same ultra-thin sections are included for enhanced contrast (Fig. 6B). To note that despite particle agglomeration, it was still possible to discern primary particles inside the cells.

Quantification of intracellular metal content by ICP-MS in the hSAEC cells was done after 24 h exposure to 50 µg/mL of SS or MS. The results showed that cells exposed to SS contained on average 15.7 pg/cell Fe and 7.7 pg/cell Mn, while MS treated cells contained on average 19.7 pg/cell Fe and 4.0 pg/cell Mn. Intracellular Ni (1.6 pg/cell) and Cr (2.3 pg/cell) were only present in the cells exposed to SS particles (Fig. 6C). Unexposed cells had levels of Fe, and Mn close to the background levels in the cell medium.

**Figure 7:**
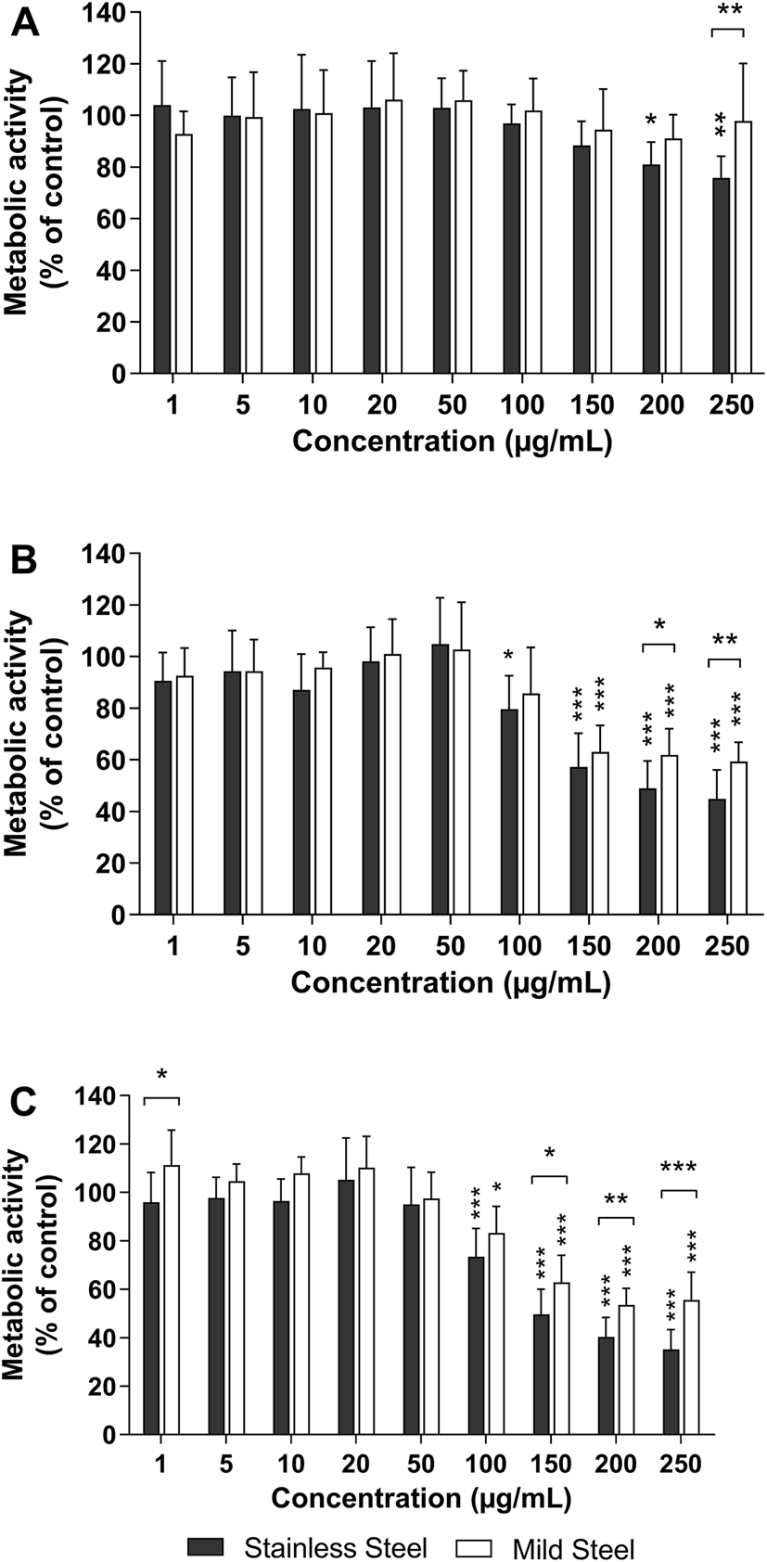
Cytotoxicity of welding particles in primary human small airway epithelial cells. Cells from three donors were exposed to stainless steel or mild steel welding particles (1–250 µg/mL) for 24 (**A**), 48 (**B**) and 72 (**C**) h. Cell viability was assessed using Alamar Blue assay and results are expressed as % metabolic activity compared to untreated cells. Results are presented as mean ± standard deviation of three independent experiments for each donor (n = 9). Statistically significant differences, as compared to the control are labelled with asterisks (* for P-value < 0.05, ** for P-value < 0.01, *** for P-value < 0.001). Figure created using GraphPad Prism version 8.3.0.

The effect of the particles on hSAEC viability was determined with the Alamar blue assay (Fig. 7). Exposure to both SS and MS welding particles reduced the metabolic activity of hSAEC, nonetheless at 24 h of exposure these effects were only evident in the cells exposed to SS particles at concentrations of 200 µg/mL or higher (Fig. 7A). With prolonged exposure time, the metabolic activity was affected at lower concentrations and SS had a stronger impact on metabolic activity in comparison to MS (Fig. 7B,C). A significant reduction in the metabolic activity of SS exposed cells was observed at concentrations equal or higher to 100 µg/ml after 48 and 72 h of exposure. In contrast, the reduction of the metabolic activity in MS exposed hSAEC was significant after 48 h at concentrations of 150 µg/mL and after 72 h at concentrations of 100 µg/mL. Likewise, significant differences between the SS and MS treatments at 24 h were only observed at the highest tested concentration (250 µg/mL). Nonetheless, with prolonged exposure times the difference between SS and MS becomes evident at lower concentrations (150 µg/mL after 72 h). There was no significant difference between primary cells derived from the three evaluated donors (data not shown).

### Stainless steel but not mild steel welding particles increase intracellular ROS production in primary human epithelial cells from the small airways

**Figure 8:**
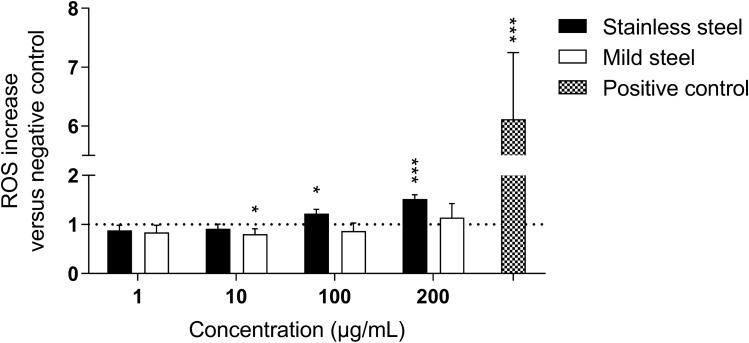
Intracellular ROS production following exposure of primary human small airway epithelial cells to welding particles. ROS production was assessed with the DCFDA reagent. hSAEC cells from three donors were exposed to stainless steel or mild steel particles (1–250 µg/mL) for 24 h. Fluorescent measurements (Ex485/Em535) were performed every 5 min over 30 min. ROS increase was calculated as mean slope per minute and normalized to the unexposed control. Tert-butyl hydroperoxide (TBP, 200 μM) was used as positive control. The data is presented as mean ± standard deviation of at least two independent experiments for each donor (n = 8). Statistically significant differences are labeled with an asterisk (* for P-value < 0.05, *** for P-value < 0.001). Figure created using GraphPad Prism version 8.3.0.

In order to further evaluate the induction of oxidative stress indicated by the reporter assay, we studied the oxidative response induced by the welding particles in primary human epithelial cells from the small airways. The generation of ROS was measured with the DCFDA reagent after 24 h exposure to SS or MS particles (Fig. 8). The results showed induction of ROS production in hSAEC exposed to SS particles at a concentration of 100 µg/mL or higher. MS particles, on the other hand, did not induce ROS production at any of the tested concentrations.

Concomitantly, the acellular production of ROS was also measured in the cell culture media used for culturing mES and hSAEC (Supplementary Fig. 3). Overall, the production of ROS was greater in the mES culture medium in comparison to hSAEC culture medium and after incubation with SS particles in comparison to MS particles. In mES medium, SS and MS particles induced ROS production at concentrations equal or higher to 100 µg/mL. In contrast, in hSAEC medium MS particles did not induce ROS production whereas SS particles induced significant ROS production at all tested concentrations with the exception of 10 µg/mL.

## Discussion

In the present study we generated stainless and mild steel welding particles using gas-metal arc welding and electrodes that are commonly used and relevant for occupational exposure to welding fumes. The particles were characterized in terms of metal composition and size distribution throughout the generation, collection processes as well as after dispersion. We first used reporter stem cells to screen for potential activated biological pathways related to development of cancer. Thereafter, we translated the observed effects in primary human small airways epithelial cells from three donors. This model is relevant since the small airways are an important site for possible injury following particle exposure—disease in this part of the lungs is key in early stages of the development of major occupational lung diseases such as COPD^26^. Evaluation of cellular localization and quantification of cellular metal content was performed in both cell systems.

We provide evidence that the metal composition in the different size fractions is homogenous for the two types of welding fumes studied. Future studies should address whether there is a higher toxicity for the smaller fraction as size-dependent toxicity (for the same mass dose) has previously been reported for welding particles^7, 21^ as well as for other metal particles *e.g.* Ag nanoparticles both in vitro and in vivo^27–29^. This is of importance for establishing occupational exposure limits (OEL) targeted at the most toxic size fraction. Currently the OEL for welding is 2.5 mg/m^3^ inorganic respirable dust (*i.e.* particle fraction with a size threshold of 4 µm).

In addition, the welding conditions are relevant both for the size distribution and chemical composition of the welding fumes, that in turn can influence toxicity. Studies indicate that at higher welding voltages the welding fumes had a higher amount of ultrafine particles^30, 31^ but overall lower lung toxicity^31^ as well as no dopaminergic neurotoxicity after inhalational exposure in rats^30^. This later finding was suggested to be related to the higher complexation and lower bioavailability of Mn in the welding fumes generated at higher voltages^30^.

The composition of the electrodes as determined by PIXE in our study was in line with the specifications from the provider. For example, PIXE analysis indicated that the SS electrode Autrod 316LSI was composed of 18% Cr, 11% Ni, 4% Mo which is comparable to 18% Cr, 12% Ni, 3% Mo from the specifications. Similar pattern was noted for the Mn composition: 1.7% Autrod 316LSI and 1.3% Aristorod 12.50 by PIXE *vs.* 1.8% Autrod 316LSI, 1.46% Aristorod 12.50 as per specifications. We show, however, an enrichment in Mn for the welding fume (approx. 26% SS, 21% MS). This was previously observed by Isaxon et al.^22^ and is likely due to the relatively low boiling point of Mn as well as to possible emissions from the welding substrate.

Our strategy to disperse the welding particles in water was suitable as it resulted in an average size of the particle agglomerates (as indicated by the DLS data) similar to the particle size of the welding agglomerates in aerosol form during generation (as indicated by the SMPS data). Inevitably, by interacting with cell medium during the cellular exposure the welding particles agglomerated to different extents, however, we could still discern individual primary particles inside the cells. The agglomeration and sedimentation processes are likely to impact the delivered as well as the cellular dose as discussed below. We report that most of the metal released in the cell media already occurs during the preparation of the sample (sonication and centrifugation) and does not increase with time which indicates that the particles have a relatively stable surface in cell medium. It is unclear what surface phenomena occur once particles are inside the cells.

A relevant finding is the striking difference in the cellular metal content in the two types of cells tested for the same nominal dose. The hSAEC had an average metal content 17 times higher for SS and 11 times higher for MS compared with the mES cells at the same exposure conditions (24 h, 50 µg/mL). This is, at least in part, mediated by the higher agglomeration and sedimentation of the welding particles in the serum free medium of the hSAEC cells compared with the serum enriched medium of the mES cells (as indicated by the DLS data). Similar findings have previously been reported for Ag nanoparticles; particle sedimentation, metal content and cytotoxicity was augmented in serum free conditions compared with serum enriched conditions in THP-1 derived macrophages^32^. In addition, the different phagocytic properties of the mES and hSAEC cells could also play a role in the observed differences. Overall, the different cellular dose could explain the lack of toxicity of the particles in the mES cells (compared with the hSAEC cells) as well as the reporter activation (Srxn1) only at the highest tested dose. As such, there could be an underestimation of the toxicity of particles when mechanistic screening is performed using reporter cells that grow in cell medium with different properties compared to the target cells. This is likely not due to the lack of sensitivity of the reporter system but rather due to the much lower delivered as well as cellular dose to the mES cells.

While the metal released in the extracellular environment could be associated with toxicity in our study, the metal released in the two types on medium was similar, which indicates that the released fraction does not bear a major role in the observed differences in toxicity. These findings point out the difficulty in comparing toxicity results across studies and in vitro models using nominal dose as a reference. The cellular dose is sometimes quantified for metal particles^32, 33^, but this is to our knowledge the first in vitro study to quantify cellular dose for welding particles.

An important finding is the activation of Srxn1 reporter by the stainless steel welding particles, which is indicative of Nrf2-dependent oxidative stress. Nrf2 is a transcription factor that translocates to the nucleus in response to oxidative stress, and activates the antioxidant response element (ARE), which in turn regulates the expression of multiple genes encoding for proteins involved in cellular adaptation to oxidative stress; Srxn1 is such an antioxidant protein^34^. Induction of the Srxn1 reporter was previously reported for metal nanoparticles containing for example copper, nickel, manganese and cobalt^35–37^, as well as for stainless steel particles with a high release of chromium in PBS^38^. In addition, activation of Nrf2 response was previously shown for stainless steel particles in a murine macrophage cell line^8^.

The activation of the Srxn1 reporter by stainless steel welding particles occurred in the absence of cytotoxicity, likewise the generation of ROS in hSAEC cells. This is important as it speaks against the observed ROS generation being an indirect result of cytotoxicity. On the contrary, the concentrations that increase ROS production at 24 h (100 µg/mL) are cytotoxic after 48 and 72 h, indicating that cytotoxicity could be, in part, mediated by oxidative stress. This is in line with other studies indicating that stainless steel particles induce oxidative stress in vitro^8, 19^. The nominal concentrations where an effect was observed in this study can be considered high, but are in the same range as previous studies have reported for welding particles with similar metal composition^7^. Other in vitro studies show effects at lower concentrations (< 20 µg/mL) but the type of particles tested were also more reactive and had a higher release of soluble Cr^8, 38^. We estimate the deposition of welding particles after a working day (8 h) at an exposure similar to OEL (2.5 mg/m^3^) in the small airways at being 0.18 µg/cm^2^/day by using the MPPD model (multiple-path particle dosimetry model v 3.04)^39^. If we add an enhancement factor of at least 10 to account for increased concentration of particles in so called deposition “hot spots”^40^, we end up with a dose of 1.8 µg/cm^2^/day. When we transform the doses used in our study from mass/volume to mass/surface area, 6 µg/mL corresponds to 1.8 µg/cm^2^. In our study we observed toxicity at doses higher than that (i.e. 100 µg/mL) and we believe these doses could be relevant considering the chronic exposure in the occupational settings for much more than just a single 8 h working day.

There was no increase in ROS generation after treatment with MS, despite increased cytotoxicity at later timepoints, which could indicate that MS and SS induce cytotoxicity via different mechanisms. One study reported reduced cell viability, increase expression of *HO-1*, but no ROS after exposure of a small airway cell line to mild steel particles (30 µg/mL)^41^. Differences observed in vitro related to the toxicity of stainless and mild steel were previously shown to be related to the presence of Cr and Ni in the stainless steel^6, 7^. However, it seems that the presence of Cr and Ni is not the ultimate player for the carcinogenic effect of welding particles; epidemiological studies indicate that exposure to mild steel (poor in Ni and Cr) is also associated with an increased risk for lung cancer^1, 9^.

Overall, our study indicates that stainless steel welding particles are more cytotoxic compared with mild steel particles and induce oxidative stress in primary human small airway epithelial cells. In addition, stainless steel particles also activate the Nrf2 dependent oxidative stress related pathways in reporter cells. Importantly, we have shown that the metal cellular content following exposure to mild- and stainless steel particles varies greatly between different cells at the same nominal dose and this could be related to differences in agglomeration/sedimentation. This emphasises the need for better understanding of the cellular dose in vitro. Finally, future studies should explore the long-term toxicity of these particles at doses that are relevant for occupational exposure in order to shed light on the molecular mechanisms behind welding-induced lung cancer.

## Supplementary Information

Supplementary Figures.
