# Supplementary material for: Toxicity of stainless and mild steel particles generated from gas–metal arc welding in primary human small airway epithelial cells
Source: Sci Rep. 2021 Nov 8;11:21846. doi: 10.1038/s41598-021-01177-7 (PMC8575907; doi:10.1038/s41598-021-01177-7)
Supplement: Supplementary file 1 — Supplementary Figures. [file 41598_2021_1177_MOESM1_ESM.pdf]

A

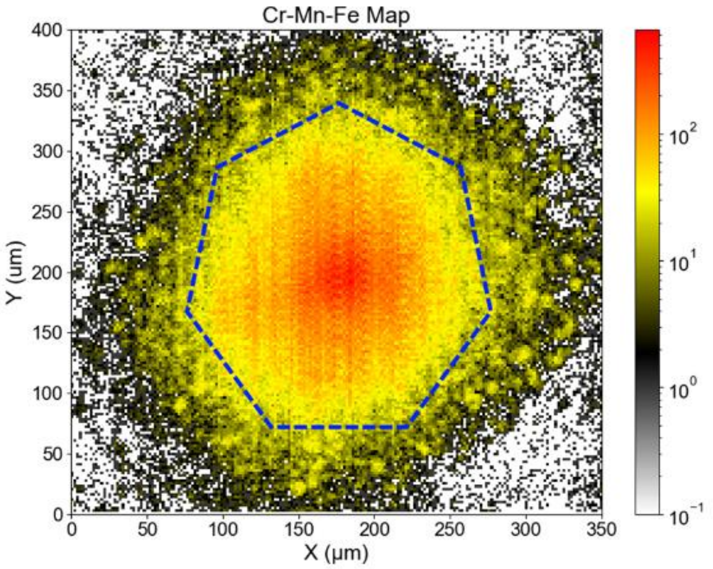

B

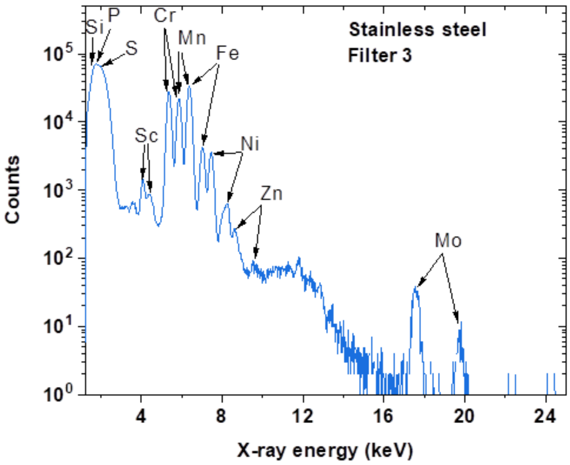

C

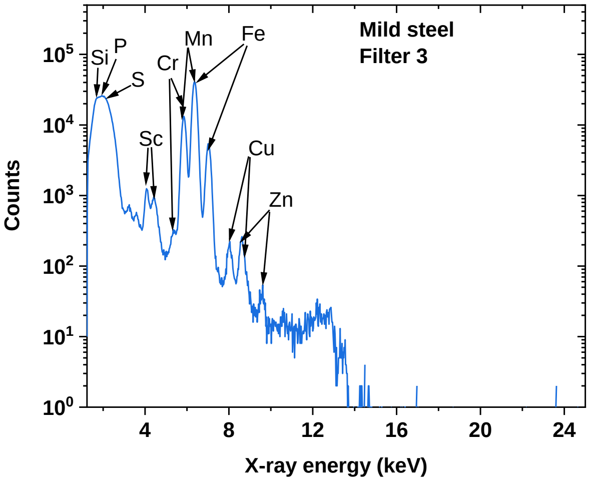

Supplementary Figure 1. (A) PIXE map of X-ray spectrum between 5.1 keV and 6.6 keV, corresponding to Cr, Mn and Fe. The blue dotted polygon indicates the region of interest selected for analysis. PIXE spectra for particles collected by impactor filter 3 for stainless steel (B) and mild steel (C). Supplementary Figure 1A was created using Python. Supplementary Figures 1B and 1C were created using Origin. OriginLab Corporation, Northampton, MA, USA.

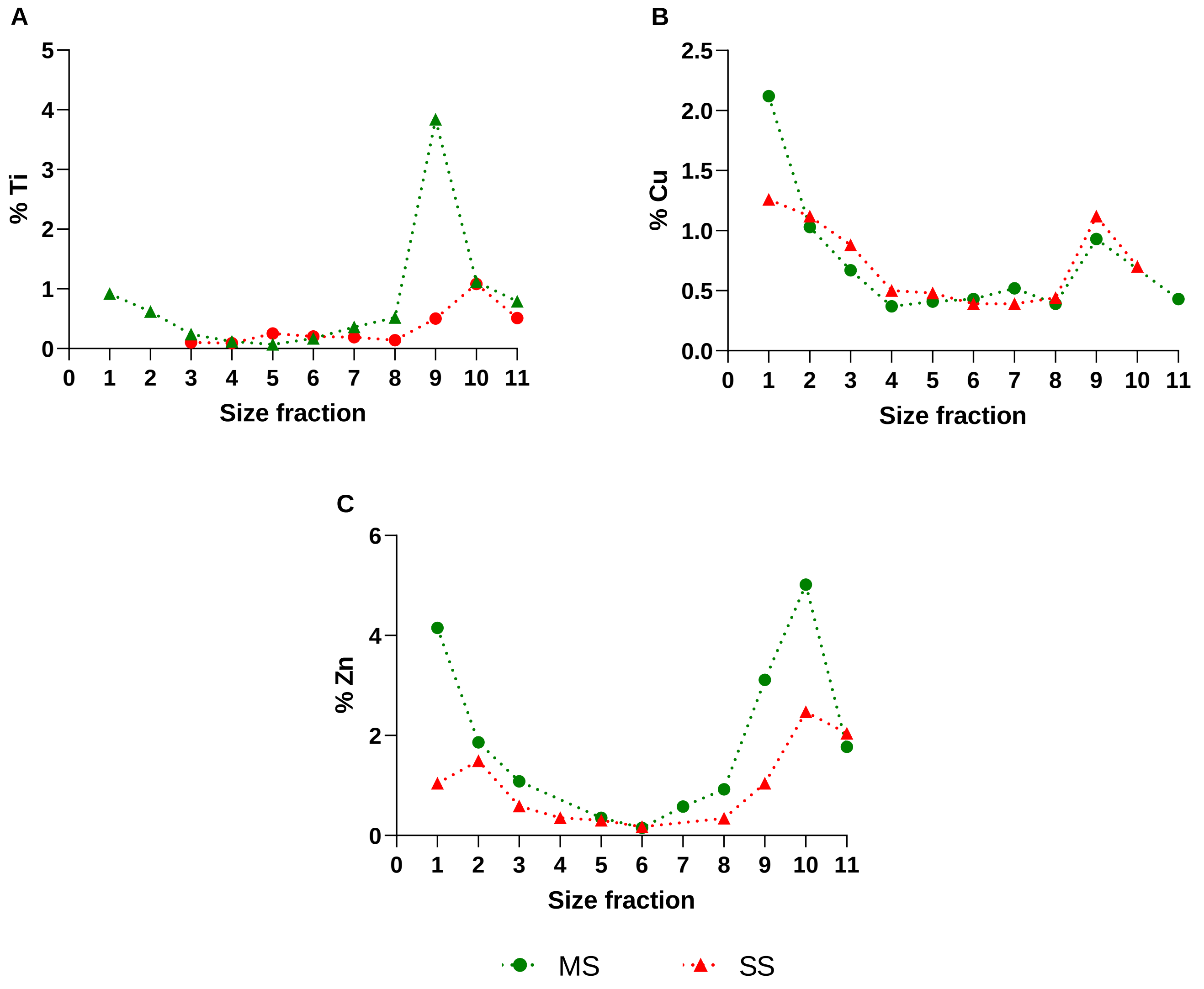

Supplementary figure 2. Characterisation of welding particles by PIXE. Quantitative metal composition was determined by PIXE on the different particle size fractions during the aerosol generation. Percentage refers to metal content which was normalized to 100% (*i.e.* it disregards the presence of oxygen). The D50 (µm) for the collection stages are the following: 1 – 0.04 µm, 2 – 0.09 µm, 3 – 0.15 µm, 4 – 0.22 µm, 5 – 0.36 µm, 6 – 0.58 µm, 7 – 0.81 µm, 8 – 1.07 µm, 9 – 1.68 µm, 10 – 2.69 µm, 11 – 4.46 µm. Figure created using GraphPad Prism version 8.3.0.

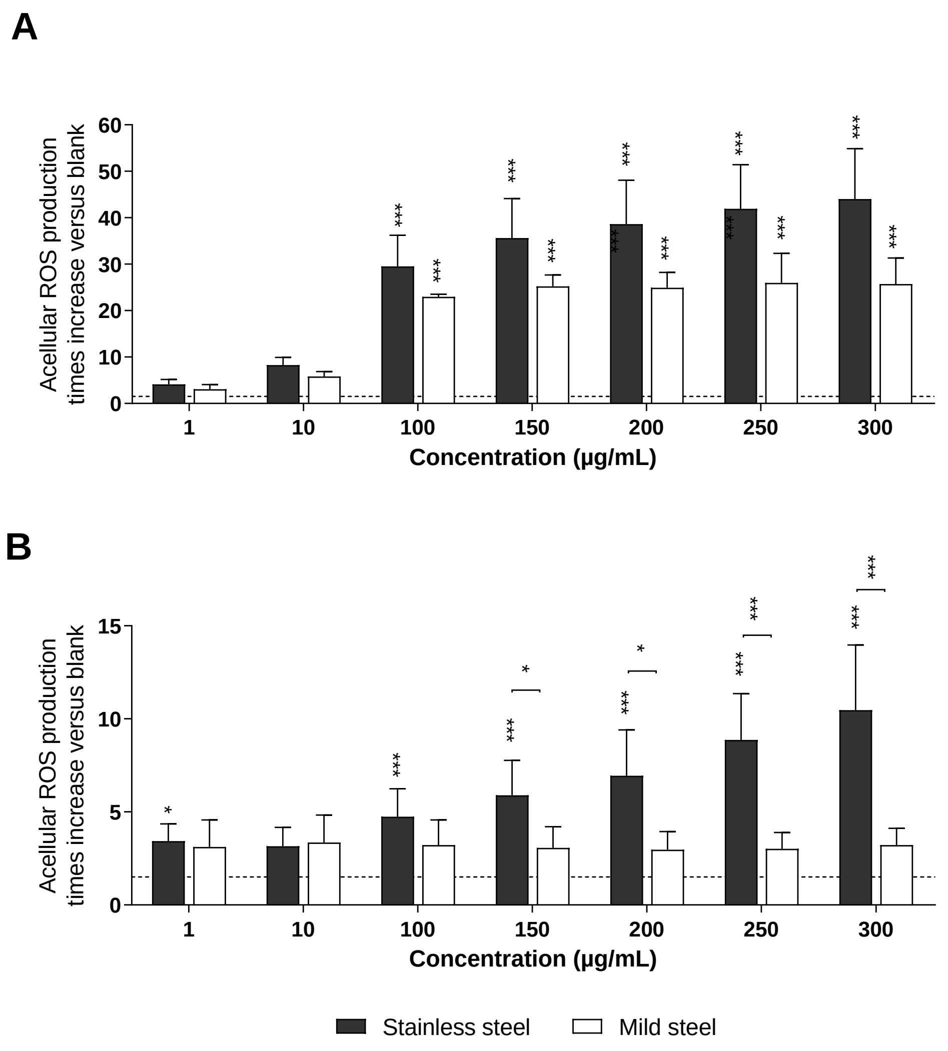

Supplementary figure 3. Acellular ROS generation after incubation with welding particles. ROS generation was assessed with the DCFDA reagent in mES (A) and hSAEC (B) culture media. Stainless steel or mild steel particles (1 – 300 µg/mL) were incubated together with de-acetylated DCFDA (DCF). the resulting fluorescent signal was measured every 5 minutes for 30 minutes. All results are expressed as times increase in the mean slope per minute of the kinetic measurement normalized against their corresponding negative control (media without particles). The data is presented as mean ± standard deviation of three independent experiments. Statistically significant differences are labeled with an asterisk (* for P-value <0.05, *** for P-value < 0.001). Figure created using GraphPad Prism version 8.3.0.
